# Revisiting the Species Delimitation Within *Amolops mantzorum* (David, 1872), with a Description of a New Subspecies (Anura, Ranidae)

**DOI:** 10.3390/ani16010055

**Published:** 2025-12-24

**Authors:** Tianyu Qian, Yu-Juan Guo, Kaidi Tian, Sheng-Chao Shi, Shun Ma, Di Zhang, Yin-Meng Hou, Feng Xie, Jian-Ping Jiang

**Affiliations:** 1 Mountain Ecological Restoration and Biodiversity Conservation Key Laboratory of Sichuan Province, Chengdu Institute of Biology, Chinese Academy of Sciences, Chengdu 610213, China; qianty@cib.ac.cn (T.Q.); guoyujuan22@mails.ucas.ac.cn (Y.-J.G.); shisc@jhun.edu.cn (S.-C.S.); mashun21@mails.ucas.ac.cn (S.M.); 2023005@htu.edu.cn (Y.-M.H.); xiefeng@cib.ac.cn (F.X.); 2 China-Croatia Belt and Road Joint Laboratory on Biodiversity and Ecosystem Services, Chengdu Institute of Biology, Chinese Academy of Sciences, Chengdu 610213, China; 3 University of Chinese Academy of Sciences, Beijing 101408, China; 4 Yellow River Water Resources Protection Institute, Zhengzhou 450004, China; tiankaidi00@163.com (K.T.); zd1874228592@163.com (D.Z.)

**Keywords:** integrative taxonomy, morphometrics, mitochondrial, systematics, torrent frog

## Abstract

Accurate species delimitation benefits detailed biological descriptions and conservation practices. In this study, we focus on a frog species distributed throughout the western edge of Sichuan Basin, China, the Sichuan torrent frog—*Amolops mantzorum*. Currently, *A. mantzorum* is recognized as comprising two subspecies—the nominate subspecies *A. m. mantzorum* and *A. m. xinduqiao*. We investigated a population from along the Yalong River, Sichuan Province, China which was previously overlooked in phylogenetic studies. Combining multiple mitochondrial genes (*16S*, *CO1*, *cytb*), we identified four distinct lineages within *A. mantzorum* using Bayesian inference and maximum likelihood phylogeny. Principal Component Analysis based on morphometrics revealed significant differences between the population from along the Yalong River and the other two subspecies for which morphological data were available. Combined with detailed morphological descriptions and comparisons, here we describe this population as the third subspecies of *A. mantzorum*—*Amolops mantzorum feiye* **ssp. nov.**

## Simple Summary

Currently, controversy about the intraspecific taxonomy of *Amolops mantzorum* remains unresolved. The Litang population was overlooked for several years in recent phylogenetic studies. We revisited this population and described the third subspecies of *A. mantzorum*, clearly dividing the four recognized lineages within *A. mantzorum* for the first time based on mitochondrial phylogeny. Our findings highlighted the importance of clear delimitation and naming of subspecies for biological conservation and taxonomic description, and provided insights for future investigations into the taxonomy of the *Amolops mantzorum* species group.

## 1. Introduction

The intraspecific taxonomy of *Amolops mantzorum* has been debated for several decades. Cai & Zhao [1] identified four genetic populations within the “*A. mantzorum* & *A. kangtingensis* complex” based on genetic distance. Two of these, exhibiting large morphometric divergence, are currently recognized as subspecies of *A. mantzorum*. *Amolops m. xinduqiao* (*A. kangtingensis* Kangding population *sensu* Cai & Zhao [1]) represents the lineage with smaller body size and a high-altitude distribution range (above 3000 m), distributed along the western slopes of Mt. Zheduo in the Yalong River Basin, whereas *A. m. mantzorum* (*A. mantzorum* Hongya and Dayi population *sensu* Cai & Zhao [1]) comprises larger-bodied, mid-to-high elevation (1200–2400 m) populations from the Dadu River Basin [2]. The two subspecies are also distinguished by different dorsal coloration patterns [2]. However, the coloration patterns can vary within a species and are thus insufficient for taxonomic diagnosis in the *A. mantzorum* group under current views. Given the small genetic divergence revealed by molecular phylogeny (i.e., Wu et al. [3]; Zeng et al. [4]), the subspecies-level classification proposed by Dufresnes & Litvinchuk [5] has been accepted in recent taxonomic systems (e.g., Tang et al. [6]; Frost [7]; Li et al. [8]).

Obviously, subspecies delimitation within *A. mantzorum* remains unresolved. Unfortunately, the *CO1* and *Cytb* sequences used by Cai & Zhao [1] were not available in their original publication. However, some evidence could be traced. Lu et al. [9] recognized an undescribed lineage from Maoxian, Sichuan Province and Wenxian, Gansu Province, designating it as “*A. mantzorum* (northern lineage)”. This population is geographically close to the Lixian and Wolong populations of *A. mantzorum* in Cai & Zhao [1]. However, the Litang population—one of the four distinct genetic populations identified by Cai & Zhao [1]—was overlooked for several years in molecular phylogeny studies [3,4,5,10].

In this study, we revisited the Litang population of *A. mantzorum*, combining specimens collected from multiple localities along the Yalong River, Sichuan Province, China. Molecular phylogeny based on mitochondrial genes suggests that this population represents a monophyletic lineage. A comprehensive morphological examination was also conducted among the recognized subspecies of *A. mantzorum*. Based on this evidence, we describe this previously overlooked lineage as a new subspecies of *A. mantzorum*.

## 2. Material and Methods

### 2.1. Samples

**Figure 1:**
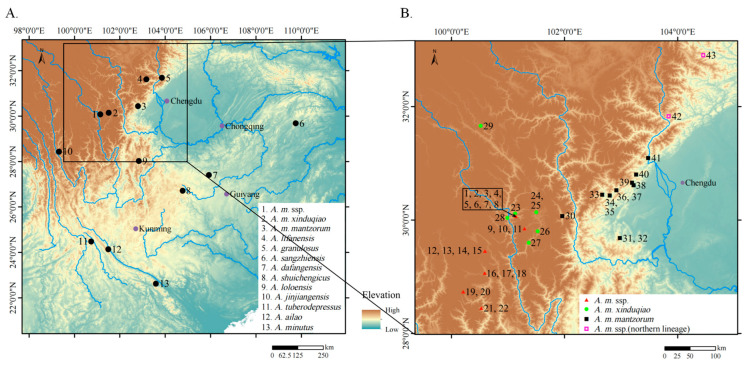
(**A**) Geographical locations of the type localities of species within the *A. mantzorum* group. (**B**) Distribution of specimens (or sequences) used in this study of *A. m.* ssp., *A. m. xinduqiao*, *A. m. mantzorum*, and “*A. m* ssp. (northern lineage)”. The numbers (**B**) beside the distribution points correspond to the IDs in Table 1.

From 2019 to 2021, 20 specimens and 2 tissue samples of the putative new subspecies were collected from Litang County, Yajiang County, Muli County, and Daocheng County in Sichuan Province (Figure 1). Additionally, 3 specimens of *A. m. xinduqiao* were collected from Yajiang County and Kangding City in Sichuan Province, and 14 specimens of *A. m. mantzorum* were collected from Baoxing County, Shimian County, Luding County and Kangding City in Sichuan Province. From the collected specimens and samples, we extracted tissues from 22 samples of the putative new subspecies, 2 of *A. m. mantzorum*, and 3 of *A. m. xinduqiao.* Muscle and/or liver tissues were preserved in 95% ethanol for molecular analyses. Specimens were fixed in formaldehyde solution and then transferred to 75% ethanol for long-term storage. All specimens were deposited in the Chengdu Institute of Biology (CIB) of Chinese Academy of Sciences (CAS). Museum specimens were also examined for morphological analyses (detailed in Appendix A).

**Table 1:** Sequences used in this study.

ID	Species	Voucher Number	Locality	*16S*	*COI*	*Cytb*
1	*Amolops mantzorum* ssp.	CIB QZ2021130	Bajiaolou Village, Bajiaolou Town, Yajiang, Sichuan	PX599057	PX599084	PX620422
2	*A. m.* ssp.	CIB QZ2021126	Bajiaolou Village, Bajiaolou Town, Yajiang, Sichuan	PX599068	PX599095	PX620433
3	*A. m.* ssp.	CIB QZ2021131	Bajiaolou Village, Bajiaolou Town, Yajiang, Sichuan	PX599075	PX599102	PX620438
4	*A. m.* ssp.	CIB QZ2021128	Bajiaolou Village, Bajiaolou Town, Yajiang, Sichuan	PX599077	PX599104	PX620440
5	*A. m.* ssp.	CIB QZ2021127	Bajiaolou Village, Bajiaolou Town, Yajiang, Sichuan	PX599079	PX599106	PX620442
6	*A. m.* ssp.	CIB YJ2019080602	Bajiaolou Village, Bajiaolou Town, Yajiang, Sichuan	PX599080	PX599107	PX620443
7	*A. m.* ssp.	CIB YJ2019080601	Bajiaolou Village, Bajiaolou Town, Yajiang, Sichuan	PX599081	PX599108	PX620444
8	*A. m.* ssp.	CIB YJ2019080603	Bajiaolou Village, Bajiaolou Town, Yajiang, Sichuan	PX599082	PX599109	PX620445
9	*A. m.* ssp.	CIB YJ202012	Nimazong Village, Zhusang Town, Yajiang, Sichuan	PX599083	PX599110	PX620446
10	*A. m.* ssp.	CIB YJ202011	Nimazong Village, Zhusang Town, Yajiang, Sichuan	PX599058	PX599085	PX620423
11	*A. m.* ssp.	CIB YJ202013	Nimazong Village, Zhusang Town, Yajiang, Sichuan	PX599059	PX599086	PX620424
12	*A. m.* ssp.	CIB LT20200712-4	Dewu Town, Litang, Sichuan	PX599060	PX599087	PX620425
13	*A. m.* ssp.	CIB LT20200712-5	Dewu Town, Litang, Sichuan	PX599061	PX599088	PX620426
14	*A. m.* ssp.	CIB LT20200712-6	Dewu Town, Litang, Sichuan	PX599062	PX599089	PX620427
15	*A. m.* ssp.	CIB LT20200712-8	Dewu Town, Litang, Sichuan	PX599063	PX599090	PX620428
16	*A. m.* ssp.	CIB DC20190905-89	Rihuo Village, Shengmu Town, Daocheng, Sichuan	PX599064	PX599091	PX620429
17	*A. m.* ssp.	CIB DC20190905-88	Rihuo Village, Shengmu Town, Daocheng, Sichuan	PX599065	PX599092	PX620430
18	*A. m.* ssp.	CIB DC20190905-91	Rihuo Village, Shengmu Town, Daocheng, Sichuan	PX599066	PX599093	PX620431
19	*A. m.* ssp.	CIB DC20190906-94	Maize Village, Mula Town, Daocheng, Sichuan	PX599067	PX599094	PX620432
20	*A. m.* ssp.	CIB DC20190906-95	Maize Village, Mula Town, Daocheng, Sichuan	PX599069	PX599096	PX620434
21	*A. m.* ssp.	CIB 2019ml0002	Xianggelila Village, Shuiluo Town, Muli, Sichuan	PX599070	PX599097	PX620435
22	*A. m.* ssp.	CIB 2019ml0001	Xianggelila Village, Shuiluo Town, Muli, Sichuan	PX599071	PX599098	PX620436
23	*A. m. xinduqiao*	CIB YJ202014	Wangxia Village, Bajiaolou Town, Yajiang, Sichuan	PX599072	PX599099	PX620437
24	*A. m. xinduqiao*	KIZ 041127	Xinduqiao Town, Kangding, Sichuan	MN953764	MN961465	-
25	*A. m. xinduqiao*	KIZ 041129	Xinduqiao Town, Kangding, Sichuan	MN953765	MN961466	-
26	*A. m. xinduqiao*	CIB GGS-PBX4-2	Geridi Village, Jiagenba Town, Kangding, Sichuan	PX599073	PX599100	-
27	*A. m. xinduqiao*	CIB GGS-SDX1-6	Laha Village, Shade Town, Kangding, Sichuan	PX599074	PX599101	-
28	*A. m. xinduqiao*	700307	Yajiang, Sichuan	-	-	KJ008410
29	*A. m. xinduqiao*	LCLH017	Luhuo, Sichuan	-	-	KJ008392
30	*A. m. mantzorum*	SYS a005360	Kangding, Sichuan	MK573807	MK568322	-
31	*A. m. mantzorum*	SYS a005336	Mt. Wawu, Hongya, Sichuan	MK573804	MK568319	-
32	*A. m. mantzorum*	SYS a005337	Mt. Wawu, Hongya, Sichuan	MK604853	MK605611	-
33	*A. m. mantzorum*	700114	Longdong Town, Baoxing, Sichuan	-	-	KJ008297
34	*A. m. mantzorum*	CIB 2020061501	Dongsheng Village, Wulong Town, Baoxing, Sichuan	PX599076	PX599103	PX620439
35	*A. m. mantzorum*	CIB 2020061502	Dongsheng Village, Wulong Town, Baoxing, Sichuan	PX599078	PX599105	PX620441
36	*A. m. mantzorum*	SYS a005370	Fengtongzhai, Baoxing, Sichuan	MK604865	MK605623	-
37	*A. m. mantzorum*	SYS a005365	Fengtongzhai, Baoxing, Sichuan	MK573808	MK568323	-
38	*A. m. mantzorum*	SCUM045825HX	Mt. Xiling Snow, Dayi, Sichuan	MN953707	MN961409	-
39	*A. m. mantzorum*	-	Mt. Xiling Snow, Dayi, Sichuan	KJ546429	KJ546429	KJ546429
40	*A. m. mantzorum*	700229	Chongzhou, Sichuan	-	-	KJ008339
41	*A. m. mantzorum*	SCUM045817HX	Wolong, Wenchuan, Sichuan	MN953706	MN961408	-
42	*A. m.* ssp. (northern lineage)	700040	Maoxian, Sichuan	-	-	KJ008277
43	*A. m.* ssp. (northern lineage)	700267	Wenxian, Gansu	-	-	KJ008360
44	*A. tuberodepressus*	SYS a003932	Mt. Wuliang, Jingdong, Yunnan	MK573800	MG991934	-
45	*A. tuberodepressus*	CIB-XM3125	Jingdong, Yunnan	KR559270	KR559270	KR559270
46	*A. ailao*	GXNU YU000004	Mt. Ailao, Xinping, Yunnan	MN650754	MN650740	MN650746
47	*A. shuichengicus*	SYS a004956	Shuicheng, Guizhou	MK604845	MK605603	-
48	*A. granulosus*	SCUM060911HX	Anxian, Sichuan	MN953681	MN961381	-
49	*A. granulosus*	20130258	Mt. Wawu, Sichuan, China	MH922934	MH922934	MH922934
50	*A. loloensis*	SYS a005346	Zhaojue, Sichuan	MK604854	MK605612	-
51	*A. loloensis*	SM-ZDTW-01	Shimian, Sichuan	KT750963	KT750963	KT750963
52	*A. jinjiangensis*	SYS a004571	Mt. Gaoligong, Yunnan	MK573801	MK568316	-
53	*A. jinjiangensis*	SCUM050435CHX	Deqing, Yunnan	EF453741	MN961403	-
54	*A. jinjiangensis*	KIZ047095	Chuxiong, Yunnan	MN953701	MN961404	-
55	*A. sangzhiensis*	CSUFT 907	Mt. Doupeng, Sangzhi, Hunan	OQ079540	OQ078905	-
56	*A. chayuensis*	SYS a007509	Baxoi, Xizang	MK573820	MK568333	-
57	*A. lifanensis*	SYS a005374	Lixian, Sichuan	MK573809	MK568324	-
58	*A. dafangensis dafangensis*	MT DF20230601002	Dafang, Guizhou	OR936315	OR924345	-
59	*A. d. dafangensis*	MT DF20230601001	Dafang, Guizhou	OR936314	OR924344	-
60	*A. d. wumengmontis*	SWU 0009561	Mt. Wumeng, Zhaotong, Yunnan, China	PX411479	PX410826	-
61	*A. d. wumengmontis*	SWU 0009562	Mt. Wumeng, Zhaotong, Yunnan, China	PX411478	PX410827	-
62	*A. minutus*	IEBR 4342	Muong La, Son La, Vietnam	-	-	MK941135
63	*A. minutus*	TBU 06	Muong La, Son La, Vietnam	-	-	MK941136
64	*A. chunganensis*	SYS a004212	Mt. Jinggang, Jinggang, Jiangxi	MK263263	MG991914	-
65	*A. nyingchiensis*	SYS a006679	Lhunze, Xizang	MK573814	MK568329	-
66	*A. cremnobatus*	ROM 14528	Khe Moi, Nghe An, Vietnam	DQ204477	-	-
67	*A. daiyunensis*	SYS a001739	Mt. Daiyun, Fujian, China	MK263243	KX507328	-
68	*A. hainanensis*	SYS a005281	Mt. Wuzhi, Hainan, China	MK263281	MG991916	-
69	*A. ricketti*	WUSTW01	Mt. Wugong, Jiangxi, China	KF956111	KF956111	KF956111
70	*A. marmoratus*	KUHE 19089	Chiang Mai, Thailand	AB211486	-	AB259738
71	*A. spinapectoralis*	ROM 37375	Ngoc Linh vicinity, Kon Tum, Vietnam	MN953726	MN961428	-
72	*A. cremnobatus*	KIZ 011621	Puhu National Reserve, Thanh Hoa, Vietnam	MN953672	MN961368	-
73	*A. wangyufani*	KIZ 014067	Zayü, Tibet, China	MN953740	MN961440	-
74	*A. cuongi*	IEBR A.5140	Tam Duong, Lai Chau, Vietnam	PX113529	-	PX119668
75	*A. cuongi*	IEBR A.5141	Tam Duong, Lai Chau, Vietnam	PX113530	-	PX119669
76	*Odorrana wuchuanensis*	LBML 5230	Libo, Guizhou, China	KU680791	KU680791	KU680791
77	*Odorrana margaretae*	HNNU 1207003	-	KJ815050	KJ815050	KJ815050

### 2.2. Molecular Analysis

We extracted genomic DNA from muscle or liver tissues using a DNA extraction kit (Tiangen Biotech (Beijing) Co., Ltd., Beijing, China). Segments of three mitochondrial genes, *16S* ribosomal RNA (*16S*), cytochrome C oxidase subunit I (*COI*), and partial cytochrome b (*Cytb*) were amplified. The *16S* primers were L3975 and H4551 [11], and the *COI* primers were Chmf4 and Chmr4 [12], and *Cytb* primers were *Cytb-L* and *Cytb-H* [13]. Amplification of the *16S*, *COI,* and *Cytb* fragments was performed in a 25 μL volume reaction: initial denaturation at 94 °C for 5 min (95 °C for *Cytb*); 34 cycles (31 cycles for *Cytb*): denaturation for 30 s at 94 °C (95 °C for *Cytb*), annealing for 30 s at 55 °C (40 s for *Cytb*), extending for 30 s at 72 °C (1 min for *Cytb*); a final extension: 7 min at 72 °C (10 min for *Cytb*). Sequencing was conducted using a 3730xl DNA sequencer by Beijing Tsingke Biotech Co., Ltd. (Beijing, China).

In addition to the 27 sequences newly obtained in this study, 50 sequences were retrieved from GenBank, including two samples of “*A. mantzorum* (northern lineage)” from Lu et al. [9], currently recognized as “*A. m.* ssp. (northern lineage)” by Tang et al. [6]. The species *Odorrana wuchuanensis* and *O. margaretae* were selected as outgroups following Tang et al. [6]. Sequences of the species in the *A. mantorum* group were selected mostly following Lyu et al. [14] and supplemented with newly described species and subspecies. To reconstruct more stable trees, we also included one or two sequences from each species group of *Amolops*. In total, 77 sequences were used for phylogenetic analysis (Table 1).

The MAFFT algorithm [15] with its default parameters in MAFFT online service (https://mafft.cbrc.jp/alignment/server/index.html [accessed on 17 December 2025]) [16] was used to align the *16S* (440 bp), *COI* (562 bp), and *Cytb* (756 bp) sequences, respectively, and AMAS [17] was used to concatenate the *16S*, *COI,* and *Cytb* sequences of each specimen. Maximum-likelihood (ML) analysis was conducted using IQ-TREE [18,19]. The support values of the phylogenetic tree were assessed using 1000 bootstrap replicates (bootstrap support value, BSV). Bayesian inference (BI) was performed in MRBAYES 3.2.1 [20] based on the best partition scheme and substitution models: GTR + G for *16S*, *CO1* first codon, and *Cytb* third codon, HKY + I for *COI* second codon and *Cytb* first codon; GTR + I + G for *CO1* third codon and *Cytb* second Codon, which were selected using ModelFinder [21] with Bayesian information criterion (BIC). Two independent runs were conducted during the BI analyses with 10,000,000 generations each, sampled every 1000 generations, with the first 25% of samples discarded as burn-in. We used a convergence diagnostic threshold of stopval = 0.01 to automatically terminate the MCMC run once convergence was achieved.

### 2.3. Morphological Analysis

Morphological characters and measurements followed Fei et al. [22], Jiang et al. [23], and Lyu et al. [14]. In total, 13 measurements were taken using slide calipers to the nearest 0.1 mm: (1) SVL, snout-vent length, from tip of snout to posterior margin of vent; (2) HL, head length, from tip of snout to the articulation of the jaw; (3) HW, head width, at the commissure of the jaws; (4) SL, snout length, from tip of snout to the anterior corner of the eye; (5) INS, internasal space, the distance between the nostrils; (6) IOS, interorbital space, the shortest distance between the upper eyelids; (7) UEW, upper eyelid width, maximum width of upper eyelid; (8) ED, eye diameter, horizontal distance from the anterior corner to the posterior corner of the eye; (9) LAHL, length of forearm and hand, from the tip of digit III to elbow joint; (10) HAL, hand length, from the proximal border of the outer palmar tubercle to the tip of digit III; (11) TL, tibia length, from the outer surface of the flexed knee to the heel; (12) TFL, length of tarsus and foot, from proximal end of tarsus to tip of toe IV; (13) FL, foot length, from proximal end of inner metatarsal tubercle to tip of toe IV.

Morphometric analyses were conducted for the putative new subspecies, *A. m. xinduqiao* and *A. m. mantzorum*. We measured specimens for numerical taxonomic analyses, including 10 adult males of the putative new subspecies, 10 adult males of *A. m. mantzorum*, and 15 adult males of *A. m. xinduqiao*. To avoid errors due to different measurement personnel, all 11 measurements were conducted by Y.-J.G. To reduce the impact of allometry, the logarithm (base 10) of the ratio of each measured value to SVL was used in the analyses. Mann–Whitney U test in SPSS (statistics 25.0) (In the “Two Independent Samples” option of the “Nonparametric Tests” option of the software, we ticked the “Mann–Whitney U” option box) was used to test for significant differences between the three subspecies of *A. mantzorum*, with significance level set at *p* < 0.05 (significant) and *p* < 0.01 (highly significant). Principal component analysis (PCA) in Origin (Pro) (Version 2024) (the “Correlation Matrix” option box in the “Analyze” option was ticked) was used to assess whether the subspecies were separated in morphometric space.

For morphological comparisons, we directly examined specimens of six species within the *Amolops mantzorum* group (detailed in Appendix A). For the remaining 12 species for which specimens were not examined, morphological data were obtained from the literature.

## 3. Results

### 3.1. Molecular Phylogeny

**Figure 2:**
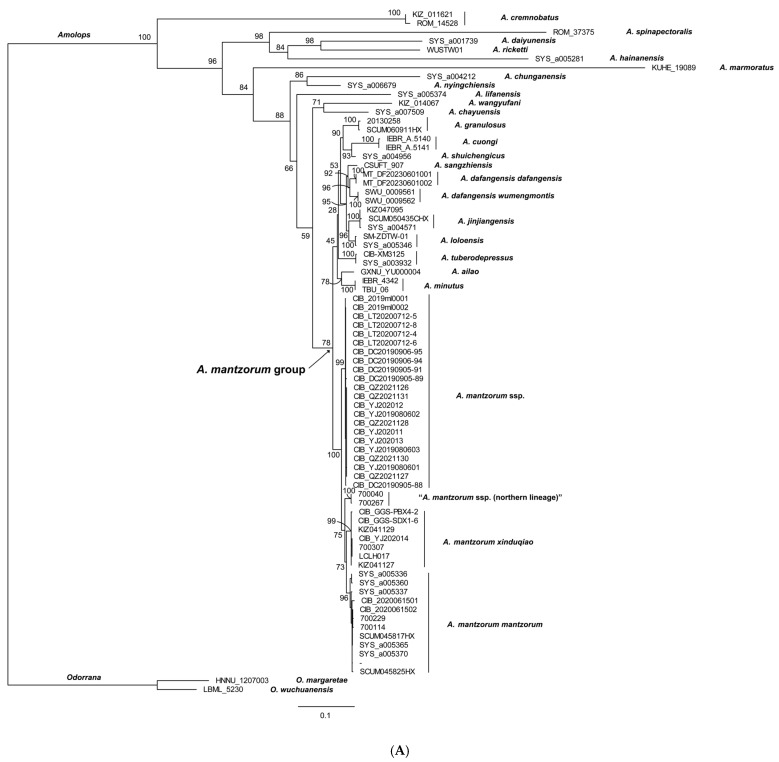

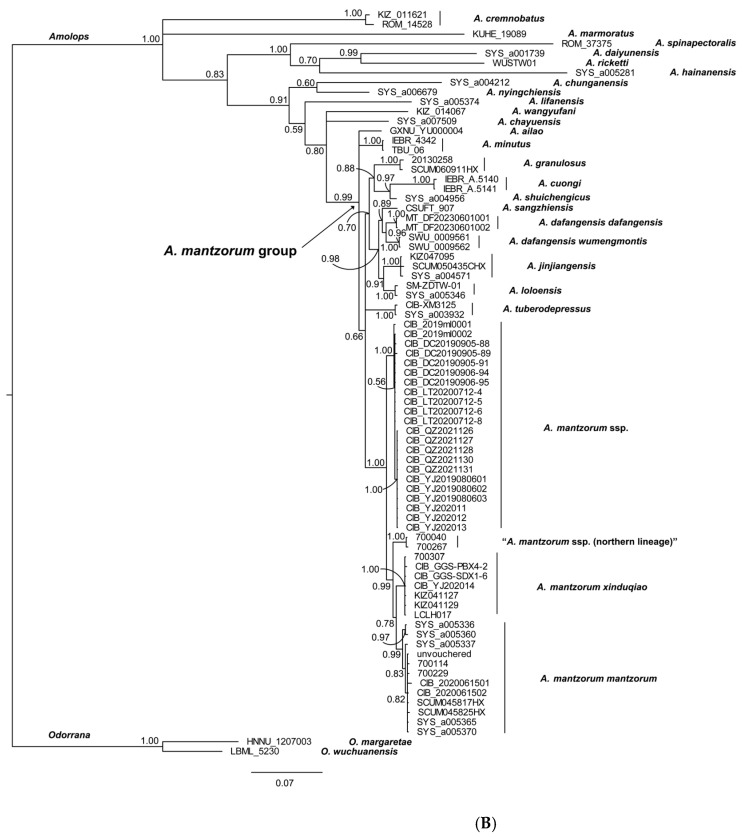
The molecular phylogenetic tree inferred from 1758 bp of *16S*-*COI-Cytb* genes (440 bp of *16S* gene, 562 bp of *COI* gene, and 756 bp of *Cytb* gene). (**A**). Maximum-likelihood tree (numbers near branches are BSV), and (**B**). Bayesian inference tree (numbers near branches are BPP), respectively. The vouchers to the right of branches correspond to the voucher numbers in Table 1.

The reconstructed phylogenies are presented in Figure 2. Both the ML and BI trees show relatively weak support values for several major clades of previously identified species groups (e.g., *A. lifanensis* of *A. mantrorum* group clustered out of its clade), indicating that the complex evolutionary history of the *A. mantzorum* group remains unresolved. A total of 22 newly collected samples (*A. mantzorum* ssp., the new lineage) from along the Yalong River clustered together as one lineage with well support (BSV 99, BPP 1.00). Samples of *A. m. xinduqiao*, *A. m. mantzorum*, and “*A. m.* ssp. (northern lineage)” each formed distinct monophyletic lineages: the *A. m. xinduqiao* lineage with strong support in both the ML and BI tree (BSV 99, BPP 1.00), the *A. m. mantzorum* lineage with strong support in both the ML and BI trees (BSV 99, BPP 0.99), and the “*A. m.* ssp. (northern lineage)” with strong support in both the BI and ML trees (BSV 100, BPP 1.00) (Figure 2). These four monophyletic lineages formed a clade with strong support in both the ML and BI trees (BSV 100), BPP 1.00) (Figure 2).

### 3.2. Morphological Comparisons Within Subspecies of A. mantzorum

Comparisons among the new lineage from along the Yalong River, *A. m. xinduqiao*, and *A. m. mantzorum* are presented herein. Morphological data on “*A. m.* ssp. (northern lineage)” are missing.

**Figure 3:**
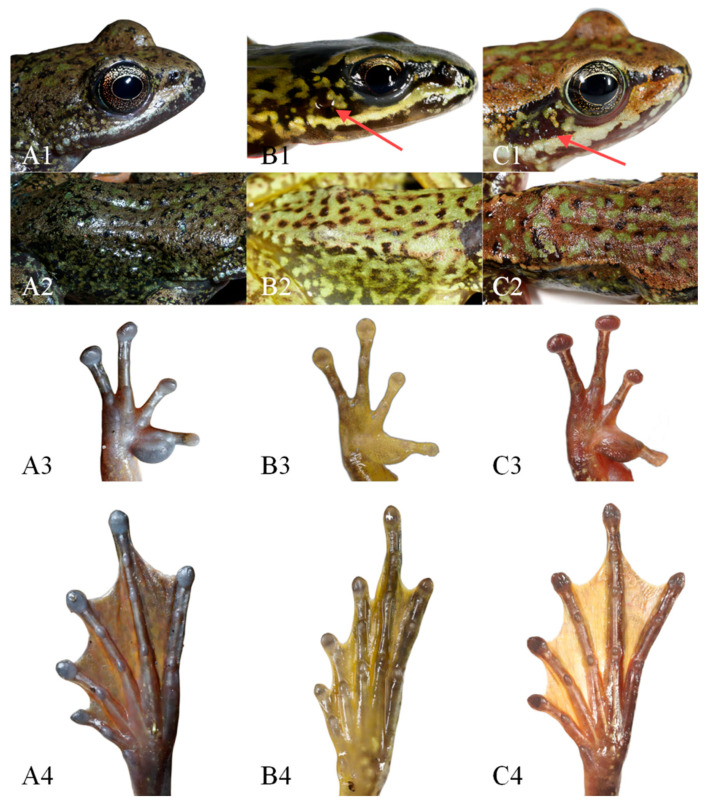
Comparisons of morphological characters of the new lineage (*Amolops mantzorum* ssp.), *A. m. xinduqiao,* and *A. m. mantzorum* in life. (**A**): *A. m.* ssp. (CIB QZ2021131); (**B**): *A. m. xinduqiao* (CIB GGS-PBX4-2); (**C**): *A. m. mantzorum* (CIB GGS-DWX2-6). (**1**): Head in dorsolateral view (red arrow points to the tympanum); (**2**): dorsolateral fold; (**3**): ventral view of hand; (**4**): ventral view of foot.

**Figure 4:**
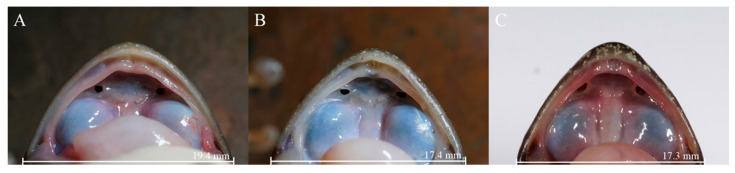
Comparisons of vomerine teeth of the new lineage (*Amolops mantzorum* ssp.) and *A. m. xinduqiao.* (**A**): *A. m.* ssp. (CIB QZ2021126, female); (**B**): *A. m.* ssp. (CIB QZ2021130, male); (**C**): *A. m. xinduqiao* (CIB GGS-PBX4-1, female).

The new lineage can be distinguished from *A. m. xinduqiao* by the absence of a tympanum (vs. small but distinct) (Figure 3), distinct and strong vomerine teeth (vs. small, two tiny rows) (Figure 4), and larger body size in adult females (SVL 56.7–63.7 mm [*n* = 10] vs. 48.5–56.6 mm [*n* = 15]) (Table 2); and from *A. m. mantzorum* by the absence of a tympanum (vs. small but distinct) (Figure 3), and smaller body size in adult males (SVL 42.3–51.2 mm [*n* = 10] vs. SVL 51.4–57.8 mm [*n* = 10]) (Table 2).

*Amolops m. xinduqiao* is distinguished from *A. m. mantzorum* by the smaller body size in adult males (SVL 40.3–46.7 mm [*n* = 15] vs. 51.4–57.8 mm [*n* = 10]) and adult females (SVL 48.5–56.6 mm [*n* = 15] vs. 59.0–72.0 mm [*n* = 10]) (Table 2).

### 3.3. Morphometric Analysis

Based on the measurements of 13 characters of the three lineages (the new lineage, *A. m. xinduqiao,* and *A. m. mantzorum*) (Table 3), the results of comparisons among them (Table 4) showed that: (1) two characters were significantly different between the new lineage and *A. m. xinduqiao*: SVL and SL/SVL; four characters were highly significantly different between them*:* UEW/SVL, ED/SVL, HAL/SVL, and TFL/SVL; (2) three characters were significantly different between the new lineage and *A. m. mantzorum*: HW/SVL, INS/SVL, and IOS/SVL, and four characters were highly significantly different between them: SVL, HL/SVL, SL/SVL, and TL/SVL; (3) one character was significantly different between *A. m. xinduqiao* and *A. m. mantzorum*: TL/SVL, and ten characters were highly significantly different between them*:* SVL, HL/SVL, HW/SVL, SL/SVL, INS/SVL, IOS/SVL, UEW/SVL, ED/SVL, HAL/SVL, and TFL/SVL.

**Figure 5:**
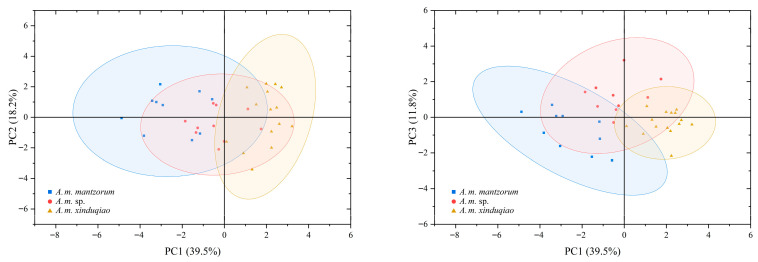
The score plot of PCA for morphological characteristics of *Amolops mantzorum* ssp., *A*. *m. xinduqiao*, and *A*. *m. mantzorum*.

In the PCA of males, the eigenvalues of the first three principal components (PC1, PC2, and PC3) were more than 1.0, and their total variation accounted for 69.5% (Table 5); therefore, the first three PCs could be used for elucidating their taxonomic significance. In the score plot of PCA (Figure 5), the plot of PC1 plus PC2 showed that the new lineage was almost separated from *A. m. xinduqiao* and *A. m. mantzorum*, though with some overlap, while the two lineages *A. m. xinduqiao* and *A. m. mantzorum* were well-separated. These differences were largely contributed by PC1 (Figure 5), to which SL and ED had higher contributions (Table 5). The plot of PC1 versus PC3 showed that the three lineages were distinctly separated from each other. These differences were contributed by combinations of PC1 and PC3 (Figure 5), of which HAL and FL had higher contributions to PC3 (Table 5). These results demonstrate that the three lineages exhibit distinct morphological differentiation.

### 3.4. Comparisons Between the New Lineage and Other Species Within the A. mantzorum Group

The morphological differences within the *A. mantzorum* group are summarized in Table 2. The new lineage can be clearly distinguished from *A. tuberodepressus*, *A. granulosus*, *A. ailao*, *A. minutus,* and *A. dafangensis* by the absence of a tympanum; differs from *A. tuberodepressus* by head width slightly larger than head length (vs. head slightly longer than broad); differs from *A. granulosus* by head width slightly larger than the head length (vs. head length slightly larger than head width), and the absence of vocal sacs (vs. present); differs from *A. ailao* by larger body size, SVL 42.3–51.2 mm in adult males and 56.7–63.7 mm in adult females (vs. 33.0–35.1 mm in males and 41.3 mm in female), head width slightly larger than head length (vs. head slightly longer than wide), and distinct and strong vomerine teeth (vs. absent); differs from *A. minutus* by larger body size, SVL 42.3–51.2 mm in adult males and 56.7–63.7 mm in adult females (vs. 29.70–36.42 mm in males and 38.47–50.22 mm in females), head width slightly larger than head length (vs. head length larger than head width), and the absence of dorsolateral folds (vs. very poorly developed); differs from *A. shuichengicus* by head width slightly larger than head length (vs. head length slightly larger than head width), and the absence of dorsolateral folds (vs. present); differs from *A. jinjiangensis* by head width slightly larger than head length (vs. head length slightly larger than head width), and the absence of dorsolateral folds (vs. present); differs from *A. loloensis* by smaller body size, SVL 42.3–51.2 mm in adult males and 56.7–63.7 mm in adult females (vs. 54.5–62.0 mm in males and 69.5–77.5 mm in females), head width slightly larger than head length (vs. head length almost equal to head width), distinct and strong vomerine teeth (vs. absent), and distinct and blunt canthus rostralis (vs. indistinct); differs from *A. sangzhiensis* by head width slightly larger than head length (vs. head length about equal to or larger than head width); differs from *A. lifanensis* by smaller body size, SVL 42.3–51.2 mm in adult males and 56.7–63.7 mm in adult females (vs. 52.0–56.0 mm in males and 61.0–79.0 mm in females), head width slightly larger than head length (vs. head length almost equal to width), and distinct and blunt canthus rostralis (vs. indistinct); differs from *A. dafangensis* by head width slightly larger than head length (vs. head length larger than head width slightly).

### 3.5. Taxonomic Account

**Figure 6:**
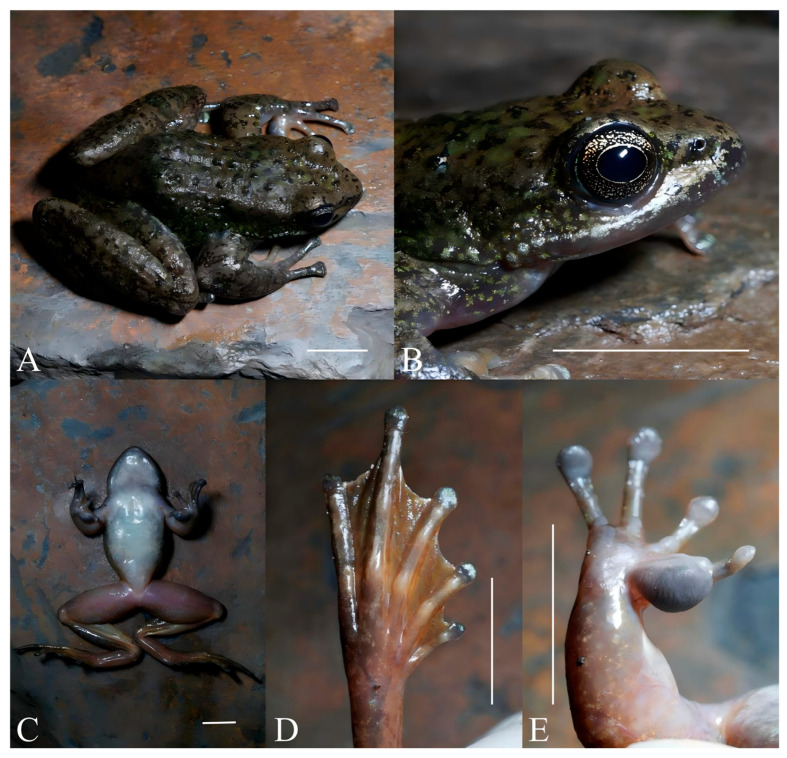
*Amolops mantzorum feiye* **ssp. nov.** (holotype, adult male, CIB QZ2021131). (**A**): Dorsolateral view; (**B**): head dorsolateral view; (**C**): ventral view; (**D**): dorsal foot; (**E**): ventral hand. Scale bars equal 10 mm.

**Figure 7:**
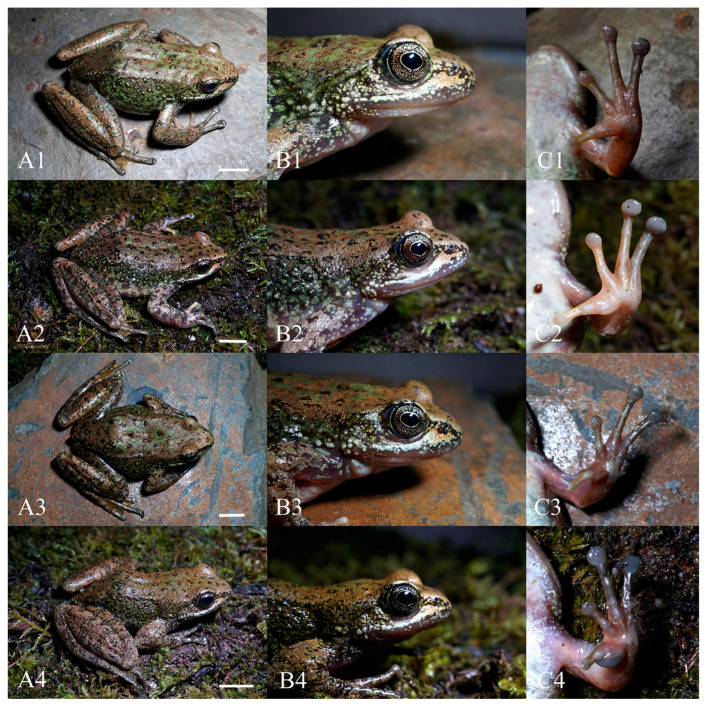
Comparisons of morphological characters of different adult individuals of *Amolops mantzorum feiye* **ssp. nov.** (the new lineage). (**A**): Dorsolateral view; (**B**): head in dorsolateral view; (**C**): ventral view of hand. (**1**): Female adult CIB QZ2021126 (SVL: 59.8 mm); (**2**): Female adult CIB QZ2021127 (SVL: 60.1 mm); (**3**): Female adult CIB QZ2021128 (SVL: 61.4 mm); (**4**): Male adult CIB QZ2021130 (SVL: 48.7 mm). Scale bar in (**A**) equals 10 mm. B and C not to scale.

*Amolops mantzorum feiye* **ssp. nov.** (Figure 6 and Figure 7; Figures S1 and S2)

Holotype. CIB QZ2021131, adult male, collected in Bajiaolou Village (30.0729° N, 101.1398° E; alt. 2841 m), Bajiaolou Town, Yajiang County, Sichuan Province, China by Shengchao Shi, Peng Yan, and Shun Ma on 20 August 2021.

Paratypes. Nineteen adult specimens (Appendix A (1)). One male (CIB QZ2021130) and three females (CIB QZ2021126, CIB QZ2021127, and CIB QZ2021128) were collected at the same time and locality as the holotype; two males (CIB YJ2019080601, CIB YJ2019080602) and one female (CIB YJ2019080603) were collected at the same locality as the holotype on 6 August 2019; two males (CIB YJ202012 and CIB YJ202013) and one female (CIB YJ202011) were collected in Nimazong Village, Zhusang Town, Yajiang County, Sichuan Province, China on 8 July 2020; four females (CIB LT20200712-4, CIB LT20200712-5, CIB LT20200712-6, CIB LT20200712-8) were collected in Dewu Town, Litang County, Sichuan Province, China on 12 July 2020; four males (CIB DC20190905-89, CIB DC20190905-91, CIB DC20190906-94, CIB DC20190906-95) and one female (CIB DC20190905-88) were collected in Daocheng County, Sichuan Province, China on 5–6 September 2019.

Diagnosis. *Amolops mantzorum feiye* **ssp. nov.** is distinguished from all other congeners by the following combination of characters: (1) moderate body size, SVL 42.3–51.2 mm in males (*n* = 10), and 56.7–63.7 mm in females (*n* = 10); (2) tympanum absent; (3) head width slightly larger than head length; (4) vomerine teeth distinct and strong; (5) male forearm relatively strong, inner side of first finger with a developed nuptial pad; (6) supernumerary tubercles below the base of fingers II, III, and IV distinct; (7) circummarginal grooves present on tips of outer three fingers, absent on finger I; (8) dorsolateral fold absent but dorsolateral fold-like glands thick and flat; (9) vocal sac absent in males.

Description of Holotype. Medium body size, SVL 47.4 mm; flat head, head width slightly larger than head length (HL/HW ratio 0.98); snout rounded, canthus rostralis distinct and blunt, loreal region concave and oblique; internasal space greater than interorbital space (INS/IOS ratio 1.28), interorbital space greater than the maximum width of upper eyelid (IOS/UEW ratio 1.52); eyes convex in dorsal view, moderate size (ED/HL ratio 0.34); tympanum absent; vomerine teeth distinct and strong; tongue deeply notched posteriorly; vocal sac absent.

Forelimbs: forearm robust, the length of forearm and hand approximately half of body length (LAHL/SVL ratio 0.47); fingers slender, all four fingers’ tips expanded into disks, and the order of finger length is I < II < IV < III; circummarginal grooves present on tips of outer three fingers, absent on finger I; subarticular tubercles distinct; supernumerary tubercles below the base of fingers II, III, and IV distinct; inner metacarpal tubercle oval, two outer metacarpal tubercles present; fringe on fingers absent; a developed nuptial pad on the inner side of the finger I.

Hindlimbs: long and thin, heels overlapping when hind limbs flexed and held perpendicular to body; all five toe tips expanded into disks; the order of toe length I < II < III < V < IV, the subarticular tubercles well-developed; entirely webbed on all toes except toe IV; inner metatarsal tubercle, outer metatarsal tubercle absent.

Skin: dorsal surface relatively smooth; supratympanic fold absent; dorsolateral fold absent but dorsolateral fold-like glands thick and flat; ventral surface smooth except for slightly flattened tubercles on basal ventral surface of thigh.

Color in life. Entire dorsal surface grayish brown with dense green stripes and black spots in the middle of the dorsal surface; upper lip grayish black, lower lip grayish white; a large area of green stripes on the lateral body, black stripes and brownish green stripes on the dorsal surface of the forelimbs, and reddish brown patches on the dorsal surface of hindlimbs; ventral surface of body opaque milky white; ventral surface of limbs red with a small amount of pale yellow spots; ventral surface of hands reddish gray; toe webs brown.

Color in preservative. Entire dorsal surface dark gray with black patches scattered; sides light grayish brown; ventral surface milky white without mottling; crotch slightly dark; dorsal surface of limbs grayish brown, with black stripes scattered, and ventral surface light gray.

Secondary sexual characters. Males obviously smaller than females; the forearm of males is relatively strong, and males have a gray nuptial pad on the inner side of finger I (Figure 7).

Variation. The dorsal surfaces of two individuals (CIB QZ2021126 and CIB YJ2019080602) from Yajiang County are light brown rather than grayish brown as in the holotype; in two individuals (CIB QZ2021127 and CIB YJ2019080603), ventral surfaces of hands are milky white rather than reddish gray as in the holotype; in three individuals (CIB QZ2021126, CIB QZ2021128 and CIB YJ2019080603), the supernumerary tubercle below the base of finger III and the lower subarticular tubercle on finger III are separate, rather than linked together as in the holotype.

In terms of body size (mean SVL), among males, the individuals from Yajiang County are larger than individuals from Daocheng County (SVL 47.8 mm [n = 6] vs. 45.2 mm [n = 4]). Among females, individuals from Yajiang County are larger than individuals from Litang County and Daocheng County (SVL 61.0 mm [n = 5] vs. 60.5 mm [n = 1] vs. 56.7 mm [n = 4]). All measurements of type series specimens are summarized in Table 6.

Etymology. The specific epithet “feiye” is named after Prof. Liang Fei and his wife Prof. Changyuan Ye, combining their first names “Fei” and “Ye”. Prof. Fei and Prof. Ye have made significant contributions to Chinese herpetology. We suggest the English common name “Feiye’s torrent frog” and the Chinese common name “费叶湍蛙 (in Chinese Pinyin: fèi yè tuān wā)”.

Distribution and ecology. At present, *A. m. feiye* **ssp. nov.** is known from Yajiang County, Daocheng County, Muli County, and Litang County in Sichuan Province (Figure 1). Specimens were found on rocks exposed to water or along the river (2800–2900 m a.s.l.), surrounded by shrubs or tussocks. The rivers crossed villages and roads where we collected these specimens.

## 4. Discussion

*Amolops m. xinduqiao* was previously recognized as “*A. kangtingensis*” in Cai & Zhao [1] (Kangding population), Lu et al. [9], and Zhang et al. [10]. However, “*A. kangtingensis*” was described based on specimens from multiple localities. Fei et al. [2] re-examined the holotype of “*A. kangtingensis*” and classified it as *A. mantzorum* (currently *A. m. mantzorum*), rendering “*A. kangtintensis*” an invalid name that should be synonymized with *A. mantzorum* (currently *A. m. mantzorum*). They proposed a new name, *A. xinduqiao* (currently *A. m. xinduqiao*) for the population studied by the above authors as distinct from *A. m. mantzorum*.

*Amolops m. mantzorum* represents the population along the Dadu River Basin according to Fei et al. [2], which also corresponds to the “*A. mantzorum* central population” of Lu et al. [9]. Samples from Hongya and Dayi represent one of the four distinct genetic populations of *A. mantzorum* identified by Cai & Zhao [1]. In our study, molecular phylogeny including samples from these two localities indicates that this population should be recognized as the nominate *A. m. mantzorum*. According to Zhang et al. [10], the distribution of *A. m. mantzorum* also includes Kangding, Luding, Yingjing, Hanyuan, Baoxing, Tianquan, and Chongzhou.

Geographically, *A. mantzorum* has a continuous distribution west of the Sichuan Basin, but four lineages can be separated based on their distribution patterns The northern lineage (“*A. m.* ssp. [northern lineage]”) is restricted to the east of the Min River. *Amolops m. mantzorum* is distributed from the Min River to the Dadu River. The new subspecies has nearly overlapping distribution with *A. m. xinduqiao* near site 23 (Figure 1) at the Yalong River, but samples were collected from different villages, and samples of *A. m. xinduqiao* were collected above 3000 m elevation, whereas samples of the new subspecies were collected at approximately 2800–2900 m. Geographical barriers formed by large rivers and different elevational preferences may have contributed to this separation of genetic lineages.

The description of the new subspecies has clarified species delimitation within *Amolops mantzorum*. The four monophyletic lineages recognized in this study are consistent with the four genetic populations revealed by Cai & Zhao [1]. Except for “*A. m.* ssp. (northern lineage)”, three of them have been described and exhibit morphological divergence. However, the delimitation of the four lineages relies solely on mitochondrial markers ([1]; this sudy). Separation in mitochondrial phylogeny is sometimes not supported by nuclear markers (e.g., Lu et al. [9]), but it can be confirmed after deeper genomic investigations [3,4]. Morphologicaly, the new subspecies can be clearly separated from the other two recognized subspecies by its distinct and strong vomerine teeth (vs. small in both *A. m. mantzorum* and *A. m. xinduqiao*), and the invisible tympanum (vs. distinct in both). The differences between *A. m. mantrozum* and *A. m. xinduqiao* are also recognized in body size.

Currently, controversy persists regarding whether these subspecies represent independent species (i.e., [28,31,32]). Given the limitations of mitochondrial genes in DNA barcoding and phylogenetic estimation [33,34,35], further studies—including a detailed description of “*A. m.* ssp. (northern lineage)”, and a more robust phylogeny based on SNP data—would benefit efforts to resolve this controversy.

## 5. Conclusions

Based on mitochondrial phylogeny and morphology, we clearly identified and described the third subspecies of *Amolops mantzorum*. The new subspecies, together with *A. m. mantzorum*, *A. m. xinduqiao*, and “*A. m.* ssp. (northern lineage)” forms four monophyletic lineages within *A. mantzorum*. Given that taxonomists do not fully agree on the placement of subspecies in these lineages, which remains controversial, future studies based on genome data and further surveys—especially on the “*A. m.* ssp. (northern lineage)”—should be prioritized.

## Figures and Tables

**Table 2:** The main morphological characters of species within the *Amolops mantzorum* group. “-” indicates no data; “*” indicates the data resource is this study.

Species	Male SVL	Female SVL	HL/HW		Dorsolateral Fold	Vocal Sac
*Amolops mantzorum* ssp.	42.3–51.2 (*n* = 10) *	56.7–63.7 (*n* = 10) *	head width slightly larger than the head length *		absent (dorsolateral fold-like glands thick and flat) *	absent *
*A. m. xinduqiao*	40.3–46.7 (*n* = 15) *	48.5–56.6 (*n* = 15)	head width slightly larger than the head length *		absent *	absent *
*A. m. mantzorum*	51.4–57.8 (*n* = 10) *	59.0–72.0 (*n* = 10)	head width slightly larger than the head length *		absent *	absent *
*A. tuberodepressus*	44.3–56.7 (*n* = 68)	60.8–71.1 (*n* = 43)	head slightly longer than broad		absent *	absent
*A. granulosus*	36.3–41.8 (*n* = 10)	51.9 (*n* = 1)	head length slightly larger than head width		absent (dorsolateral fold-like glands present)	present
*A. ailao*	33.0–35.1 (*n* = 6)	41.3 (*n* = 1)	head slightly longer than wide		absent (dorsolateral folds formed by series of glands present)	absent
*A. minutus*	29.70–36.42 (*n* = 8)	38.47–50.22 (*n* = 5)	head length more than head width		very poorly developed	well developed
*A. shuichengicus*	34.6–39.6 (*n* = 8)	48.5–55.5 (*n* = 4)	head length slightly larger than head width		present	absent
*A. jinjiangensis*	43.0–53.8 (*n* = 22)	53.6–66.4 (*n* = 20)	head length slightly larger than the head width		present	absent
*A. loloensis*	54.5–62.0 (*n* = 19)	69.5–77.5 (*n* = 20)	head length almost equal to the head width		absent	absent
*A. sangzhiensis*	40.3–40.9 (*n* = 3)	52.6–57.7 (*n* = 3)	head length about equal to or larger than head width		absent (developed a series of elongated glands forming an incomplete line)	absent
*A. lifanensis*	52.0–56.0 (*n* = 5)	61.0–79.0 (*n* = 10)	head length almost equal to the head width		-	absent
*A. dafangensis*	43.2–46.8 (*n* = 3)	-	head length larger than head width slightly		weak formed by series of glands	absent
**Species**	**Vomerine Teeth**		**Tympanum**	**Canthus Rostralis**	**Type Locality**	**Data Resource**
*Amolops mantzorum* ssp.	distinct and strong *		absent *	distinct and blunt *	Bajiaolou Village, Bajiaolou Town, Yajiang, Sichuan *	This study
*A. m. xinduqiao*	small, two tiny rows *		small but distinct *	distinct *	Xinduqiao Town, Kangding, Sichuan	Fei et al. [2]
*A. m. mantzorum*	weak and small		small but distinct	slightly distinct	Baoxing, Sichuan	Fei et al. [24]
*A. tuberodepressus*	patches in two short, oblique rows		distinct	distinct	Mt. Wuliang, Jingdong, Yunnan	Liu and Yang [25]
*A. granulosus*	two short lines		small but distinct	distinct	Maoxian, Sichuan	Fei et al. [24]
*A. ailao*	absent		distinct	distinct	Xinping, Yunnan	Tang et al. [6]
*A. minutus*	strongly developed		well developed	-	Ho Thau Village, Ho Thau Commune, Tam Duong District, Lao Chai, Vietnam	Orlov and Ho [26]
*A. shuichengicus*	present		indistinct	distinct and blunt	Shuicheng, Guizhou	Lyu et al. [14]
*A. jinjiangensis*	oblique arrangement		indistinct	distinct	Benzilan Town, Deqin, Yunnan	Su et al. [27]
*A. loloensis*	absent		small and indistinct	indistinct	Zhaojue, Sichuan	Fei et al. [24]
*A. sangzhiensis*	present		indistinct and small	-	Mt. Doupeng, Sangzhi, Hunan	Qian et al. [28]
*A. lifanensis*	developed		indistinct	indistinct	Lixian, Sichuan	Fei et al. [24]; Fei [29]
*A. dafangensis*	present		distinct	-	Dafang, Guizhou	Li et al. [30]

**Table 3:** Measurements (in mm; mean ± SD (range)) of the male type series of the new lineage (*Amolops mantzorum* ssp.), *A. m. xinduqiao*, and *A. m. mantzorum*.

Characters	*A. m.* ssp. (*n* = 10)	*A. m. xinduqiao* (*n* = 15)	*A. m. mantzorum* (*n* = 10)
SVL	46.8 ± 2.9 (42.3–51.2)	43.3 ± 2.2 (40.3–46.7)	54.1 ± 2.3 (51.4–57.8)
HL	15.0 ± 1.1 (12.9–16.4)	14.2 ± 1.2 (12.4–15.9)	15.8 ± 1.1 (13.8–17.3)
HW	16.0 ± 1.3 (14.2–18.0)	15.3 ± 0.9 (13.5–16.6)	17.6 ± 1.2 (15.9–20.0)
SL	6.5 ± 0.4 (6.0–7.3)	6.5 ± 0.3 (5.9–7.1)	6.8 ± 0.4 (6.1–7.6)
INS	5.3 ± 0.2 (5.0–5.7)	4.8 ± 0.3 (4.3–5.5)	5.5 ± 0.6 (4.4–6.5)
IOS	4.2 ± 0.2 (3.9–4.6)	4.1 ± 0.3 (3.6–4.6)	4.2 ± 0.4 (3.6–5.0)
UEW	3.4 ± 0.3 (2.8–3.8)	3.6 ± 0.4 (3.1–4.8)	3.8 ± 0.7 (3.1–5.2)
ED	5.1 ± 0.5 (4.1–6.1)	5.9 ± 0.4 (5.2–6.3)	5.6 ± 0.4 (4.9–6.3)
LAHL	23.6 ± 1.8 (20.0–26.4)	22.3 ± 1.0 (21.0–24.7)	28.0 ± 1.5 (26.0–31.8)
HAL	13.5 ± 1.6 (10.4–16.2)	14.0 ± 0.8 (13.0–15.9)	16.2 ± 1.2 (14.3–18.6)
TL	27.4 ± 1.3 (25.7–29.2)	25.6 ± 1.5 (22.8–28.6)	33.9 ± 1.4 (31.6–36.5)
TFL	39.3 ± 2.6 (35.4–43.7)	34.2 ± 1.7 (31.8–37.9)	45.9 ± 2.9 (41.4–51.5)
FL	27.7 ± 2.2 (25.5–32.0)	25.0 ± 1.2 (23.7–27.6)	30.5 ± 2.2 (25.7–32.6)

**Table 4:** Morphometric comparisons among males of the new lineage (*Amolops mantzorum* ssp.)*, A. m. xinduqiao*, and *A. m. mantzorum*. *p*-values are results from Mann–Whitney U tests on each character between subspecies. (* *p*-value < 0.05; ** *p*-value < 0.01).

Character	*A. m.* ssp. vs. *A. m. xinduqiao*	*A. m.* ssp. vs. *A. m. mantzorum*	*A. m. xinduqiao* vs. *A. m. mantzorum*
log_10_ (SVL)	0.010722 *	0.000157 **	0.000032 **
log_10_ (HL/SVL)	0.244071	0.005159 **	0.000202 **
log_10_ (HW/SVL)	0.052204	0.034294 *	0.000385 **
log_10_ (SL/SVL)	0.01982 *	0.00194 **	0.000032 **
log_10_ (INS/SVL)	0.911664	0.010165 *	0.009132 **
log_10_ (IOS/SVL)	0.107697	0.012611 *	0.000584 **
log_10_ (UEW/SVL)	0.006567 **	0.596701	0.009132 **
log_10_ (ED/SVL)	0.000385 **	0.449692	0.000032 **
log_10_ (LAHL/SVL)	0.134214	0.226476	0.911664
log_10_ (HAL/SVL)	0.001294 **	0.173617	0.003921 **
log_10_ (TL/SVL)	0.374799	0.00194 **	0.012555 *
log_10_ (TFL/SVL)	0.002282 **	0.405679	0.001568 **
log_10_ (FL/SVL)	0.134214	0.173617	0.541749

**Table 5:** Factor loadings of the first three principal components among *Amolops mantzorum* ssp., *A. m. xinduqiao*, and *A. m. mantzorum*.

Characters	PC1	PC2	PC3
log_10_ (HL/SVL)	0.3447	−0.1823	0.0592
log_10_ (HW/SVL)	0.3467	0.1735	−0.0007
log_10_ (SL/SVL)	0.3917	0.0735	0.1646
log_10_ (INS/SVL)	0.2514	−0.0279	0.3357
log_10_ (IOS/SVL)	0.3651	0.1035	0.3069
log_10_ (UEW/SVL)	0.3432	0.0282	−0.2217
log_10_ (ED/SVL)	0.3817	−0.0123	−0.0292
log_10_ (LAHL/SVL)	0.0741	0.5217	−0.3162
log_10_ (HAL/SVL)	0.2176	0.3498	−0.4449
log_10_ (TL/SVL)	−0.1416	0.4890	−0.0373
log_10_ (TFL/SVL)	−0.2731	0.4042	0.3064
log_10_ (FL/SVL)	0.0085	0.3490	0.5652
Eigenvalue	4.7367	2.1870	1.4104
Percentage of variance (%)	39.4727	18.2248	11.7533
Cumulative percentage (%)	39.4727	57.6976	69.4509

**Table 6:** Measurements (in mm; mean ± SD [range]) of adult specimens (type series) of *Amolops mantzorum feiye* **ssp. nov.**

Characters	Males (*n* = 10)	Ratio to the SVL (%)	Females (*n* = 10)	Ratio to the SVL (%)
SVL	46.8 ± 2.9 (42.3–51.2)		60.3 ± 2.0 (56.7–63.7)	
HL	15.0 ± 1.1 (12.9–16.4)	32.0 ± 1.9 (28.1–34.1)	17.5 ± 0.9 (15.6–18.8)	29.0 ± 1.3 (25.8–30.2)
HW	16.0 ± 1.3 (14.2–18.0)	34.1 ± 1.2 (32.1–35.8)	19.6 ± 1.1 (17.8–21.2)	32.5 ± 1.1 (31.2–34.6)
SL	6.5 ± 0.4 (6.0–7.3)	14.0 ± 1.0 (12.1–15.7)	7.8 ± 0.6 (7.0–9.1)	13.0 ± 0.8 (12.0–14.3)
INS	5.3 ± 0.2 (5.0–5.7)	11.3 ± 0.9 (10.4–13.0)	6.1 ± 0.3 (5.6–6.8)	10.2 ± 0.6 (9.2–11.1)
IOS	4.2 ± 0.2 (3.9–4.6)	9.0 ± 0.6 (8.3–10.2)	4.5 ± 0.4 (4.3–5.5)	7.5 ± 0.6 (6.7–8.7)
UEW	3.4 ± 0.3 (2.8–3.8)	7.4 ± 0.8 (5.8–8.3)	3.8 ± 0.3 (3.4–4.4)	6.3 ± 0.6 (5.5–7.2)
ED	5.1 ± 0.5 (4.1–6.1)	10.9 ± 1.3 (9.0–14.1)	6.0 ± 0.2 (5.7–6.4)	9.9 ± 0.5 (9.4–10.9)
LAHL	23.6 ± 1.8 (20.0–26.4)	50.4 ± 1.8 (47.3–52.6)	28.8 ± 1.1 (27.0–30.2)	47.8 ± 1.7 (44.5–49.4)
HAL	13.5 ± 1.6 (10.4–16.2)	28.9 ± 2.5 (24.7–31.8)	15.6 ± 1.3 (13.2–17.8)	25.9 ± 2.4 (21.5–28.9)
TL	27.4 ± 1.3 (25.7–29.2)	58.6 ± 1.7 (56.2–61.8)	32.9 ± 0.6 (32.1–33.8)	54.6 ± 1.5 (52.0–56.9)
TFL	39.3 ± 2.6 (35.4–43.7)	84.0 ± 2.1 (80.1–87.3)	45.9 ± 2.5 (39.6–48.8)	76.1 ± 4.7 (64.3–80.7)
FL	27.7 ± 2.2 (25.5–32.0)	59.3 ± 2.7 (54.5–63.9)	32.4 ± 2.2 (27.3–35.1)	53.8 ± 4.5 (44.4–59.3)

## Data Availability

All data are provided in the main text or Supplementary Materials.

## Supplementary Materials

The following supporting information can be downloaded at https://www.mdpi.com/article/10.3390/ani16010055/s1, Figure S1: Dorsolateral view of *Amolops mantzorum feiye* **ssp. nov.** (the new lineage); Figure S2: Head dorsolateral view of *Amolops mantzorum feiye* **ssp. nov.** (the new lineage).

## Appendix A. Specimens Examined

(1) *Amolops mantzorum feiye* **ssp. nov.** (*n* = 20): Sichuan Province, China:

Yajiang County: Bajiaolou Town: Bajiaolou Village (type locality): CIB QZ2021131 (holotype), CIB QZ2021130, CIB QZ2021126– 28, and CIB YJ2019080601–03;

Yajiang County: Zhusang Town: Nimazong Village: CIB YJ202011–13;

Daocheng County: Shengmu Town: Rihuo Village: CIB DC20190905-88–89, and CIB DC20190905-91;

Daocheng County: Mula Town: Maize Village: CIB DC20190906-94–95;

Litang County: Dewu Town: CIB LT20200712-4–6 and CIB LT20200712-8.

(2) *A. m. xinduqiao* (*n* = 18)*:* Sichuan Province, China:

Kangding City: Jiagenba Town, Geridi Village: CIB GGS-PBX4-1–2;

Kangding City: Xinduqiao Town (type locality): CIB 80I0692 (holotype), CIB 80I0704, CIB 80I0725, CIB 80I0729–30, CIB 80I0737–44, CIB 80I0747, and CIB 80I0775;

Yajiang County: Shuiluo Town: Wangxia Village: CIB YJ202014.

(3) *A. m. mantzorum (n* = 14): Sichuan Province, China:

Baoxing County (type locality): Wulong Town: Dongsheng Village: CIB 2020061501–02, 04–05;

Shimian County: Caoke Town: CIB GGS-CKX1-2–3;

Shimian County: Caoke Town: Heping Village: CIB GGS-CKX5-1–3;

Luding County: Tianba Town: CIB GGS-TBX4-1 and CIB GGS-TBX4-2;

Luding County: Dewei Town: Zawei Village: CIB GGS-DWX2-6;

Kangding City: CIB GGS-GZ2-01–02.

(4) *A. loloensis* (*n* = 7): Sichuan Province, China:

Baoxing County: Wulong Town: Dongsheng Village: CIB 2020061506–07;

Shimian County: Caoke Town: Heping Village: CIB GGS-CKX5-5;

Luding County: Tianba Town: CIB GGS-TBX4-2 and CIB GGS-TBX4-7–9.

(5) *A. jinjiangensis* (*n* = 10): Yunnan Province, China:

Deqin County (type locality): CIB 5334220036, CIB 5334220039–40, CIB 5334220042, CIB 5334220093–97, and CIB 5334220146.

(6) *A. tuberodepressus* (*n* = 7): Yunnan Province, China:

Jingdong County (type locality): CIB JDA01–02, CIB JD20230725001–02, CIB JDH01–03.

## References

[1] Cai H.X., Zhao E.M. (2008). The validity of four *Amolops* species in Hengduan Mountains China. Sichuan J. Zool..

[2] Fei L., Ye C.Y., Wang Y.F., Jiang K. (2017). A new species of the genus *Amolops* (Anura: Ranidae) from high-altitude Sichuan, southwestern China, with a discussion on the taxonomic status of *Amolops kangtingensis*. Zool. Res..

[3] Wu Y.H., Yan F., Stuart B.L., Prendini E., Suwannapoom C., Dahn H.A., Zhang B.L., Cai H.X., Xu Y.B., Jiang K. (2020). A combined approach of mitochondrial DNA and anchored nuclear phylogenomics sheds light on unrecognized diversity, phylogeny, and historical biogeography of the torrent frogs, genus *Amolops* (Anura: Ranidae). Mol. Phylogenet. Evol..

[4] Zeng Z.C., Liang D., Li J.X., Lyu Z.T., Wang Y.Y., Zhang P. (2020). Phylogenetic relationships of the Chinese torrent frogs (Ranidae: *Amolops*) revealed by phylogenomic analyses of AFLP-Capture data. Mol. Phylogenet. Evol..

[5] Dufresnes C., Litvinchuk S.N. (2022). Diversity, distribution and molecular species delimitation in frogs and toads from the Eastern Palaearctic. Zool. J. Linn. Soc..

[6] Tang S.J., Sun T., Liu S., Luo S.D., Yu G.H., Du L. (2023). A new species of cascade frog (Anura: Ranidae: *Amolops*) from central Yunnan, China. Zool. Lett..

[7] Frost D.R. Amphibian Species of the World, Version 6.2, an Online Reference.

[8] Li P.Y., Li J.Y., Zhang W., Yi X.B., Yuan Z.Y., Huang J.K., Liu X.L. (2025). Integrated evidence reveals a new subspecies of *Amolops* Cope, 1865 (Anura, Ranidae) from northeastern Yunnan, China. ZooKeys.

[9] Lu B., Bi K., Fu J.Z. (2014). A phylogeographic evaluation of the *Amolops mantzorum* species group: Cryptic species and plateau uplift. Mol. Phylogenet. Evol..

[10] Zhang C.H., Yuan S.Q., Xia Y., Zeng X.M. (2015). Species delimitation of *Amolops kangtingensis*. Sichuan J. Zool..

[11] Simon C., Frati F., Beckenbach A., Crespi B., Liu H., Flook P. (1994). Evolution, weighting and phylogenetic utility of mitochondrial gene sequences and a compilation of conserved polymerase chain reaction primers. Ann. Entomol. Soc. Am..

[12] Che J., Chen H.M., Yang J.X., Jin J.Q., Jiang K., Yuan Z.Y., Murphy R.W., Zhang Y.P. (2012). Universal COI primers for DNA barcoding amphibians. Mol. Ecol. Resour..

[13] Wu X.Y. (2015). Genetic Structure and Phylogeography of *Amolops wuyiensis* Based on Mitochondrial DNA Cytb Gene. Master’s Thesis.

[14] Lyu Z.T., Zeng Z.C., Wan H., Yang J.H., Li Y.L., Pang H., Wang Y.Y. (2019). A new species of *Amolops* (Anura: Ranidae) from China, with taxonomic comments on *A. liangshanensis* and Chinese populations of *A. marmoratus*. Zootaxa.

[15] Kuraku S., Zmasek C.M., Nishimura O., Katoh K. (2013). aLeaves facilitates on-demand exploration of metazoan gene family trees on MAFFT sequence alignment server with enhanced interactivity. Nucleic Acids Res..

[16] Katoh K., Rozewicki J., Yamada K.D. (2019). MAFFT online service: Multiple sequence alignment, interactive sequence choice and visualization. Brief. Bioinfom..

[17] Borowiec M.L. (2016). AMAS: A fast tool for alignment manipulation and computing of summary statistics. PeerJ.

[18] Nguyen L., Schmidt H.A., von Haeseler A., Minh B.Q. (2015). IQ-TREE: A Fast and Effective Stochastic Algorithm for Estimating Maximum-Likelihood Phylogenies. Mol. Biol. Evol..

[19] Wong T.K.F., Ly-Trong N., Ren H., Banos H., Roger A.J., Susko E., Bielow C., De Maio N., Goldman N., Hahn M.W. (2025). IQ-TREE 3: Phylogenomic Inference Software Using Complex Evolutionary Models. Ecoevorxiv.

[20] Ronquist F., Teslenko M., Mark P., Ayres D.L., Darling A., Höhna S., Larget B., Liu L., Suchard M.A., Huelsenbeck J.P. (2012). MrBayes 3.2: Efficient Bayesian phylogenetic inference and model choice across a large model space. Syst. Biol..

[21] Kalyaanamoorthy S., Minh B.Q., Wong T.K.F., von Haeseler A., Jermiin L.S. (2017). ModelFinder: Fast model selection for accurate phylogenetic estimates. Nat. Method..

[22] Fei L., Hu S.Q., Ye C.Y., Huang Y.Z. (2009). Fauna Sinica Amphibia, Vol. 2: Anura.

[23] Jiang K., Wang K., Yan F., Xie J., Zhou D.H., Liu W.L., Jiang J.P., Li C., Che J. (2016). A new species of the genus *Amolops* (Amphibia: Ranidae) from southeastern Tibet, China. Zool. Res..

[24] Fei L., Hu S.Q., Ye C.Y., Huang Y.Z. (2009). Fauna Sinica Amphibia, Vol. 3: Anura Ranidae.

[25] Liu W.Z., Yang D.T. (2000). A new species of *Amolops* (Anura: Ranidae) from Yunnan, China, with a discussion of karyological diversity in *Amolops*. Herpetologica.

[26] Orlov N.L., Ho C.T. (2007). Two new species of cascade ranids of *Amolops* genus (Amphibia: Anura: Ranidae) from Lai Chau Province (northwest Vietnam). Russian J. Herpetol..

[27] Su C.Y., Yang D.T., Li S.M. (1986). A new species of *Amolops* from the Hengduan Shan Mountains. Acta Herpetol. Sin..

[28] Qian T.Y., Xiang J.J., Jiang J.P., Yang D.D., Gui J. (2023). A new species of the *Amolops mantzorum* group (Anura: Ranidae: *Amolops*) from northwestern Hunan Province, China. Asian Herpetol. Res..

[29] Fei L. (2020). Atlas of Amphibians in China (Field Edition).

[30] Li S.Z., Liu J., Ke X.C., Cheng G., Wang B. (2024). A new species of *Amolops* (Amphibia, Anura, Ranidae) from Guizhou Province, China. ZooKeys.

[31] Wang K., Zhang D.R., Hou S.B., Wu Y.H. (2022). Annual Review: Taxonomic Changes of Herpetofauna from China in 2022. AmphibiaChina.

[32] AmphibiaChina The Database of Chinese Amphibians. http://www.amphibiachina.org/.

[33] Chan K.O., Hertwig S.T., Neokleous D.N., Flury J.M., Brown R.M. (2022). Widely used, short 16S rRNA mitochondrial gene fragments yield poor and erratic results in phylogenetic estimation and species delimitation of amphibians. BMC Ecol. Evol..

[34] Hupało K., Copilaș-Ciocianu D., Leese F., Weiss M. (2022). Morphology, nuclear SNPs and mate selection reveal that COI barcoding overestimates species diversity in a Mediterranean freshwater amphipod by an order of magnitude. Cladistics.

[35] Vences M., Miralles A., Dufresnes C. (2024). Next-generation species delimitation and taxonomy: Implications for biogeography. J. Biogeogr..

