# Supplementary material for: Revisiting the Species Delimitation Within Amolops mantzorum (David, 1872), with a Description of a New Subspecies (Anura, Ranidae)
Source: Animals (Basel). 2025 Dec 24;16(1):55. doi: 10.3390/ani16010055 (PMC12784691; doi:10.3390/ani16010055)
Supplement: Supplementary file 1 [file animals-16-00055-s001.zip › animals-4005350-supplementary.pdf]

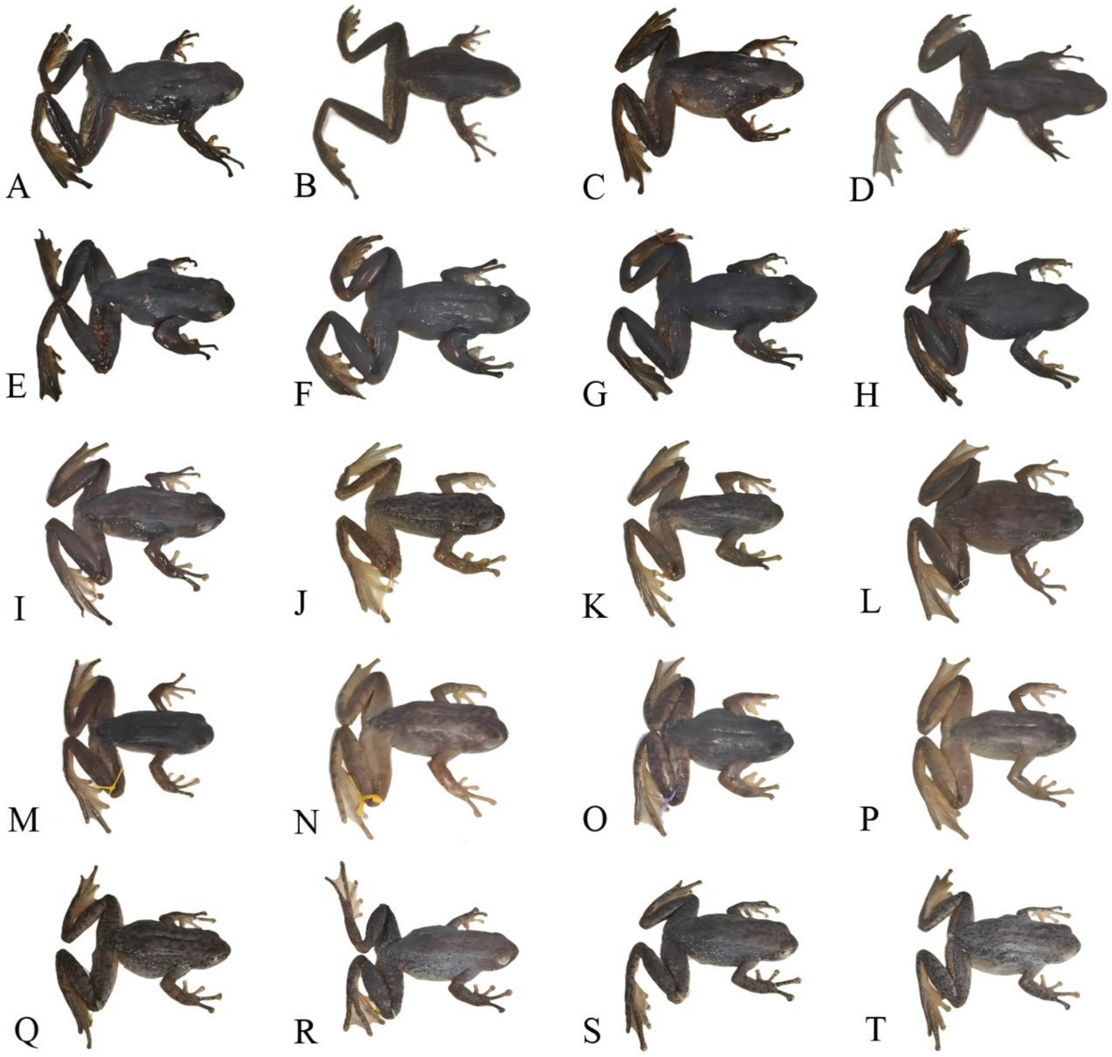

Figure S1. Dorsolateral view of *Amolops mantzorum feiye* ssp. nov. (the new lineage). A: CIB QZ2021126, B: CIB QZ2021127, C: CIB QZ2021128. D: CIB QZ2021130, E: CIB QZ2021131, F: CIB YJ2019080601, G: CIB YJ2019080602, H: CIB YJ2019080603, I: CIB YJ202011, J: CIB YJ202012, K: CIB YJ202013, L: CIB DC20190905-88, M: CIB DC20190905-89, N: DC20190905-91, O: CIB DC20190906-94, P: DC20190906-95, Q: CIB LT20200712-4, R: CIB LT20200712-5, S: CIB LT202007126, T: CIB LT20200712-8.

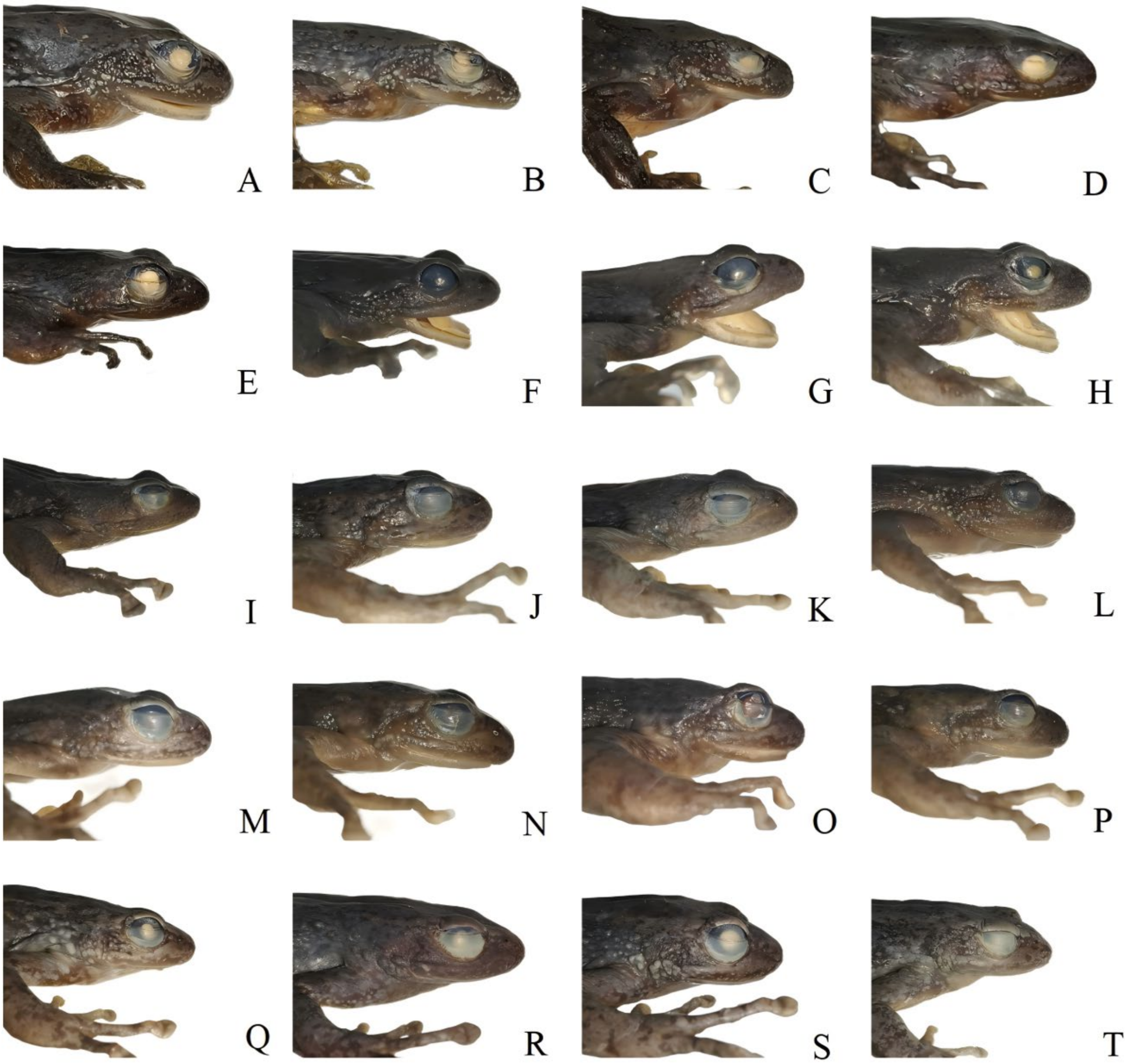

Figure S2. Head dorsolateral view of *Amolops mantzorum feiye* ssp. nov. (the new lineage). A: CIB QZ2021126, B: CIB QZ2021127, C: CIB QZ2021128. D: CIB QZ2021130, E: CIB QZ2021131, F: CIB YJ2019080601, G: CIB YJ2019080602, H: CIB YJ2019080603, I: CIB YJ202011, J: CIB YJ202012, K: CIB YJ202013, L: CIB DC20190905-88, M: CIB DC20190905-89, N: DC20190905-91, O: CIB DC20190906-94, P: DC20190906-95, Q: CIB LT20200712-4, R: CIB LT20200712-5, S: CIB LT202007126, T: CIB LT20200712-8.
